# ﻿A new species of the genus *Leptobrachella* Smith 1925 (Amphibia, Anura, Megophryidae) from Guangxi, China

**DOI:** 10.3897/zookeys.1178.106038

**Published:** 2023-09-01

**Authors:** Wei-Cai Chen, Wan-Xiao Peng, Peng Li, Gui-Dong Yu

**Affiliations:** 1 Key Laboratory of Environment Change and Resources Use in Beibu Gulf Ministry of Education, Nanning Normal University, Nanning 530001, Guangxi, China; 2 Guangxi Key Laboratory of Earth Surface Processes and Intelligent Simulation, Nanning Normal University, Nanning 530001, Guangxi, China; 3 Guangxi Forest Inventory and Planning Institute, Nanning 530011, Guangxi, China

**Keywords:** Bioacoustics, molecular analyses, morphological characters, sympatric species, taxonomy

## Abstract

A new species of *Leptobrachella*, *L.wumingensis***sp. nov.**, was described from the Damingshan National Nature Reserve, Wuming District, Nanning City, Guangxi, China based on morphological, molecular and bioacoustic data. Phylogenetic analysis of 16S mtDNA fragments revealed that the new species is closely related to *L.damingshanensis*. Uncorrected *p*-distances between the new species and all homologous DNA sequences available for the 16S gene of *Leptobrachella* are greater than 7.1%. Morphologically, *L.wumingensis***sp. nov.** differs from its congeners in several ways, including a medium body size (SVL 26.0–26.7 mm in males, 30.6–34.8 mm in females), lack of toe webbing and lateral fringes, shagreened and granular dorsal surface, pale brown dorsum with darker brown markings, iris bicolored, with the upper half copper and fading to silver in the lower half, and the presence of small irregular black spots and tangerine tubercles on the flanks. Furthermore, we found the new species to have two types of advertisement calls and relatively high dominant frequencies, making it distinct from its congeners.

## ﻿Introduction

The genus *Leptobrachella* Smith, 1925 is distributed in northeastern India, Southeast Asia, Vietnam, Thailand, Myanmar, Malaya, Borneo and Natuna Island (Frost 2023). The species diversity of *Leptobrachella* has been drastically underestimated due to its small body size, conserved morphology and high degree of sympatry (Chen et al. 2018; Chen et al. 2021a, 2021b). However, by utilizing morphological, molecular and bioacoustic data, numerous new *Leptobrachella* species have been described in recent decades (Frost 2023). The genus now comprises 99 species, of which 37 were described in the last five years (Frost 2023). In China, there are 39 *Leptobrachella* species, and 27 of them were described in the past five years, mostly occurring in southern and western China (AmphibiaChina 2023), indicating an underestimation of the diversity of *Leptobrachella*.

Between 2019 and 2023, we conducted surveys at the Damingshan National Nature Reserve (DMS), Wuming District, Nanning City, Guangxi, China. We collected nine specimens (hereafter DMS specimens) of *Leptobrachella* that significantly differed from the sympatric species, *L.damingshanensis* Chen, Yu, Cheng, Meng, Wei, Zhou & Lu, 2021 and other congeners in morphological characters, including body size and color pattern as well as molecular data. The uncorrected *p*-distances between 16S mtDNA fragments of DMS specimens and all homologous sequences of *Leptobrachella* available in GenBank were greater than 7.1%. Additionally, the bioacoustic data of the DMS specimens distinguished them from *L.damingshanensis* and other advertisement calls available in the genus *Leptobrachella*. Given the unique morphological characters, the relatively high degree of mtDNA divergence, and the distinct bioacoustics data, we describe the DMS specimens as a new species of *Leptobrachella*.

## ﻿Material and methods

**Figure 1.**
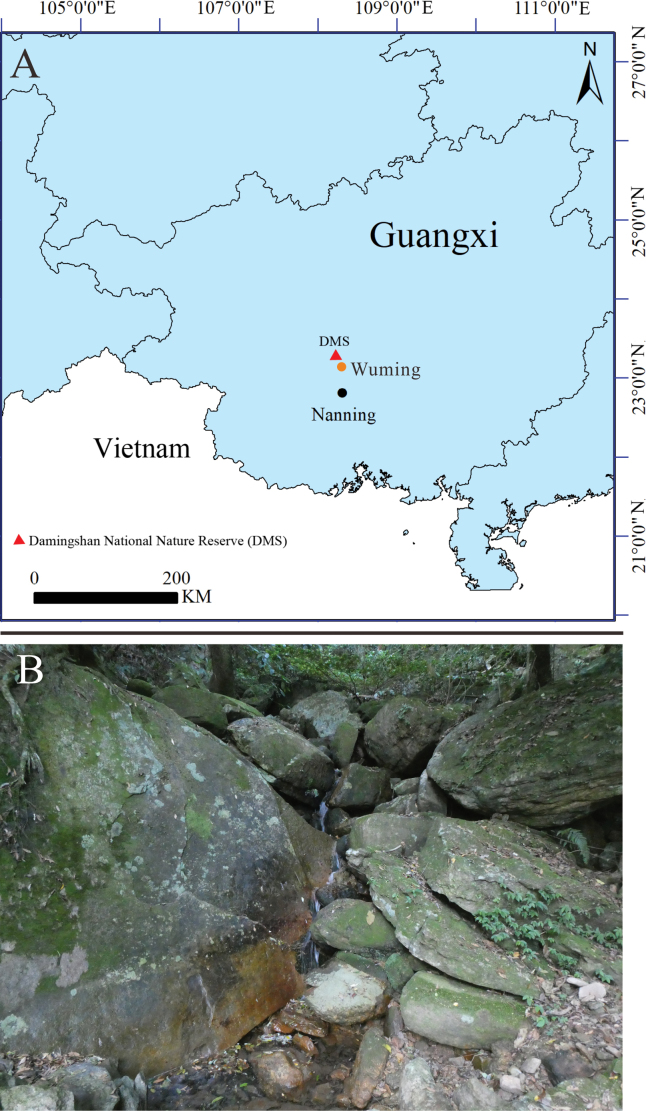
Type locality of *L.wumingensis* sp. nov. (**A**) and its habitat (**B**).

Nine *Leptobrachella* specimens were collected from the Damingshan National Nature Reserve, Wuming District, Nanning City, Guangxi, China (Fig. 1). After fixing the specimens in 10% formalin, they were stored in 75% ethanol and deposited at Nanning Normal University (NNU). Muscle tissue was taken before fixing and stored in 100% ethanol for DNA isolation. We measured all specimens to the nearest 0.1 mm using a digital caliper, following Fei et al. (2009) and Rowley et al. (2016), which included the snout-vent length (SVL) , head length from tip of snout to rear of jaws (HL) , head width at commissure of jaws (HW) , snout length from tip of snout to the anterior eye (SNT) , diameter of the exposed portion of eye (ED) , interorbital distance (IOD) , internarial distance (IN) , horizontal diameter of tympanum (TD) , distance from anterior edge of the tympanum to posterior corner of eye (TED) , manus length from tip of third finger to base of inner palmar tubercle (ML) , forelimb length from elbow to the tip of third finger (FLL) , thigh length from vent to knee (THL) , tibia length with flexed hindlimb (TIB) , pes length from tip of fourth toe to base of inner metatarsal tubercle (PL) , and maximum diameter of femoral gland (FEM). Sex was determined by the calls of males or gonadal inspection. Comparative morphological data were collected from the literature (Suppl. material 1: table S1) and from the collected specimens (Suppl. material 1: table S2).

Genomic DNA was isolated from muscle tissue using Tiangen Biotech Co. Ltd. tissue extraction kits (Beijing, China). The primer pairs 16Sar_L 5’-CGCCTGTTTAC CAAAAACAT-3’ and 16Sbr_H 5’-CCGGTCTGAACTCAGATCACGT-3’ (Palumbi et al. 1991) were used to amplify and sequence mitochondrial 16S rRNA gene fragments (~530 bp). The polymerase chain reaction (PCR) amplification was carried out in a 20 μl reaction volume involving the following steps: an initial denaturation step at 95 °C for 3 min, followed by 35 cycles of denaturation at 95 °C for 35 s, annealing at 58 °C for 40 s, and extension at 72 °C for 40 s, and a final extension step at 72 °C for 10 min. The fragments were sequenced using an ABI Prism 3730 automated DNA sequencer (Applied Biosystems, USA), and the newly obtained sequences were submitted to GenBank (accession numbers OM935575–OM935578, OR194551–OR194553).

Phylogenetic relationships were inferred using 16S mtDNA fragments through maximum likelihood (ML) and Bayesian inference (BI) analyses. Table 1 provides detailed information on the sequences used in the molecular analyses, which were aligned using the Clustalw module in MEGA v. 7 (Kumar et al. 2016) with default settings. The best-fit models of evolution (GTR+I+G) were determined using MrModeltest v. 2.3 (Nylander 2004) with Akaike and Bayesian information criteria. ML analyses were conducted on the CIPRES science gateway, estimating the proportion of invariable sites from the data and performing 1000 bootstrap pseudo replicates (https://www.phylo.org/portal2) (Miller et al. 2010). BI was performed using MrBayes v. 3.2 (Ronquist et al. 2012), with two independent runs and four Markov Chain Monte Carlo simulations for 30 million iterations. Trees were sampled every 1000 generations, with the first 25% of trees discarded as burn-in. Node values were considered well supported if ML bootstrap supports (BS) were ≥ 70% and Bayesian posterior probabilities (PP) were ≥ 0.95. Outgroups used were *Leptobrachiumhuashen* and *Xenophrysglandulosa* following Chen et al. (2018). Uncorrected *p*-distances were calculated based on 16S gene fragments using Mega v. 7 with default settings.

**Table 1.** DNA sequences used in this study. ‘*’ represents type locality.

ID	Species	Locality	Voucher no.	GenBank No. 16S rRNA
1	*L.wumingensis* **sp. nov.**	Wuming County, Guangxi, China*	NNU 00283	OM935575
2	*L.wumingensis* **sp. nov.**	Wuming County, Guangxi, China*	NNU 00284	OM935576
3	*L.wumingensis* **sp. nov.**	Wuming County, Guangxi, China*	NNU 00285	OM935577
4	*L.wumingensis* **sp. nov.**	Wuming County, Guangxi, China*	NNU 00286	OM935578
5	*L.wumingensis* **sp. nov.**	Wuming County, Guangxi, China*	NNU 01058	OR194551
6	*L.wumingensis* **sp. nov.**	Wuming County, Guangxi, China*	NNU 01059	OR194552
7	*L.wumingensis* **sp. nov.**	Wuming County, Guangxi, China*	NNU 01086	OR194553
8	*L.aerea*	Quang Binh, Vietnam	ZFMK 86362	JN848409
9	*L.alpina*	Caiyanghe, Yunnan, China	KIZ 049024	MH055867
10	*L.applebyi*	Phong Dien Nature Reserve, Thua Thien-Hue, Vietnam	KIZ 010701	MH055947
11	*L.arayai*	Borneo, Malaysia*	AE 100/S9	DQ642119
12	*L.ardens*	Kon Ka Kinh National Park, Gia Lai, Vietnam*	ZMMU-NAP-06099	MH055949
13	*L.aspera*	Huanglianshan Nature Reserve, Lyuchun, Yunnan, China*	SYS a007743	MW046199
14	*L.baluensis*	Sabah, Borneo, Malaysia*	SP 21604	LC056792
15	*L.bashaensis*	Basha Nature Reserve, Guizhou, China*	GIB 196404	MW136295
16	*L.bidoupensis*	Bidoup-Nui Ba National Park, Lam Dong, Vietnam*	ZMMU-A-4797-01454	MH055945
17	*L.bijie*	Bijie City, Guizhou, China*	SYS a007313	MK414532
18	*L.botsfordi*	Lao Cai, Vietnam*	AMS R 176540	MH055952
19	*L.bourreti*	Mao’er Shan, Guangxi, China	KIZ 019389	MH055869
20	*L.brevicrus*	Sarawak, Borneo, Malaysia*	ZMH A09365	KJ831302
21	*L.chishuiensis*	Guizhou, China*	CIB CS20190518047	MT117053
22	*L.crocea*	Thua Thien-Hue, Vietnam	ZMMU-NAP-02274	MH055955
23	*L.damingshanensis*	Wuming County, Guangxi, China*	NNU 202103281	MZ145229
24	*L.damingshanensis*	Wuming County, Guangxi, China*	NNU 202103282	MZ145230
25	*L.damingshanensis*	Wuming County, Guangxi, China*	NNU 202103283	MZ145231
26	*L.dong*	Tongdao County, Hunan Province, China*	CIB SSC1758	OP764529
27	*L.dorsospina*	Yushe Forest Park, Shuicheng, Guizhou, China*	SYS a004961	MW046194
28	*L.dringi*	Borneo, Malaysia*	KUHE:55610	AB847553
29	*L.eos*	Phongsaly, Laos*	MNHN 2004.0274	JN848452
30	*L.feii*	Yunnan, China*	KIZ048894	MT302634
31	*L.firthi*	Kon Tum, Vietnam*	AMS: R 176524	JQ739206
32	*L.flaviglandulosa*	Xiaoqiaogou Nature Reserve, Yunnan, China*	KIZ 016072	MH055934
33	*L.fritinniens*	Danum Valley Field Center, Sabah, Malaysia	FMNH 244800	MH055971
34	*L.fuliginosa*	Phetchaburi, Thailand	KUHE:20197	LC201988
35	*L.gracilis*	Bukit Kana, Sarawak, Malaysia	FMNH 273682	MH055972
36	*L.graminicola*	Mount Pu Ta Leng, Lao Cai, Vietnam*	VNMN 010909	MZ224649
37	*L.hamidi*	Borneo, Malaysia*	KUHE 17545	AB969286
38	*L.heteropus*	Peninsular, Malaysia	KUHE 15487	AB530453
39	*L.isos*	Gia Lai, Vietnam*	AMS R 176480	KT824769
40	*L.itiokai*	Gunung Mulu National Park, Sarawak, Malaysia*	KUHE:55897	LC137805
41	*L.jinshaensis*	Lengshuihe Nature Reserve, Jinsha County, Guizhou, China*	CIB JS20200516001	MT814014
42	*L.juliandringi*	Sarawak, Borneo, Malaysia*	KUHE 17557	LC056784
43	*L.kajangensis*	Tioman, Malaysia*	LSUHC:4439	LC202002
44	*L.kalonensis*	Binh Thuan, Vietnam*	IEBR A.2014.15	KR018114
45	*L.kecil*	Cameron, Malaysia*	KUHE:52439	LC202003
46	*L.khasiorum*	Meghalaya, India*	SDBDU 2009.329	KY022303
47	*L.korifi*	Doi Inthanon, Thailand*	KUHE 19134	LC741033
48	*L.laui*	Wutongshan, Shenzhen city, China*	SYS a001507	KM014544
49	*L.liui*	Wuyi Shan, Fujian, China*	ZYCA907	MH055908
50	*L.macrops*	Dak Lak, Vietnam*	AMS R177663	KR018118
51	*L.maculosa*	Ninh Thuan, Vietnam*	AMS: R 177660	KR018119
52	*L.mangshanensis*	Manghan, Hunan, China*	MSZTC201703	MG132198
53	*L.maoershanensis*	Mao’er Shan, Guangxi, China	KIZ 07614	MH055927
54	*L.marmorata*	Borneo, Malaysia*	KUHE 53227	AB969289
55	*L.maura*	Borneo, Malaysia	SP 21450	AB847559
56	*L.melanoleuca*	Kapoe, Ranong, Thailand	KIZ 018031	MH055967
57	*L.melica*	Ratanakiri, Cambodia*	MVZ 258198	HM133600
58	*L.minima*	Doi Phu Fa, Nan, Thailand	KIZ 024317	MH055852
59	*L.mjobergi*	Sarawak, Borneo, Malaysia*	KUHE 47872	LC056787
60	*L.murphyi*	Doi Inthanon, Chiang Mai, Thailand*	KIZ 031199	MZ710523
61	*L.nahangensis*	Tuyen Quang, Vietnam*	ROM 7035	MH055853
62	*L.namdongensis*	Thanh Hoa, Vietnam*	VNUF A.2017.95	MK965390
63	*L.neangi*	Veal Veng District, Pursat, Cambodia*	CBC 1609	MT644612
64	*L.niveimontis*	Yongde County, Yunnan, China*	KIZ 028276	MT302620
65	*L.nyx*	Ha GiangProv., Vietnam*	AMNH A 163810	DQ283381
66	*L.oshanensis*	Emei Shan, Sichuan, China*	Tissue ID: YPX37492	MH055896
67	*L.pallida*	Lam Dong, Vietnam*	UNS 00510	KR018112
68	*L.parva*	Mulu National Park, Sarawak, Malaysia*	KUHE:55308	LC056791
69	*L.pelodytoides*	NA	TZ 819	AF285192
70	*L.petrops*	Ba Vi National Park, Ha Tay, Vietnam	ROM 13483	MH055901
71	*L.picta*	Borneo, Malaysia	UNIMAS 8705	KJ831295
72	*L.pluvialis*	Lao Cai, Vietnam*	MNHN:1999.5675	JN848391
73	*L.puhoatensis*	Nghe An, Vietnam*	VNMN 2016 A.22	KY849586
74	*L.purpurus*	Yunnan, China*	SYS a006530	MG520354
75	*L.purpuraventra*	Guizhou, China*	SYS a007281	MK414517
76	*L.pyrrhops*	Loc Bac, Lam Dong, Vietnam*	ZMMU-A-4873-00158	MH055950
77	*L.rowleyae*	Da Nang City, Vietnam*	ITBCZ2783	MG682552
78	*L.sabahmontanus*	Borneo, Malaysia*	BORNEENSIS 12632	AB847551
79	*L.shangsiensis*	Shangsi County, Guangxi, China*	NHMG1401032	MK095460
80	*L.shimentaina*	Shimentai Nature Reserve, Guangdong, China*	SYS a004712	MH055926
81	*L.shiwandashanensis*	Fangcheng City, Guangxi, China*	NNU202103146	MZ326691
82	*L.sinorensis*	Mae Hong Son, Thailand*	KUHE 19809	LC741034
83	*L.sola*	Gunung Stong, Kelantan, Malaysia	KU RMB20973	MH055973
84	*L.suiyangensis*	Guizhou, China*	GZNU 20180606005	MK829649
85	*L.sungi*	Vinh Phuc, Vietnam*	ROM 20236	MH055858
86	*L.tadungensis*	Dak Nong, Vietnam*	UNS 00515	KR018121
87	*L.tengchongensis*	Yunnan, China*	SYS a004598	KU589209
88	*L.tuberosa*	Kon Ka Kinh National Park, Gia Lai, Vietnam*	ZMMU-NAP-02275	MH055959
89	*L.ventripunctata*	Wenlong, Yunnan, China	KIZ 013621	MH055824
90	*L.wuhuangmontis*	Pubei County, Guangxi, China*	SYS a003486	MH605578
91	*L.wulingensis*	Hunan, China*	CSUFT194	MT530316
92	*L.yeae*	Mount Emei, Sichuan, China*	CIBEMS20190422HLJ1-6	MT957019
93	*L.yingjiangensis*	Yunnan, China*	SYS a006532	MG520351
94	*L.yunkaiensis*	Guangdong, China*	SYS a004663	MH605584
95	*L.yunyangensis*	Yunyang County, Chongqing, China*	GZNU 20210629001	OL800366
96	*L.zhangyapingi*	Chiang Mai, Thailand*	KIZ 07258	MH055864
97	*Leptobrachiumhuashen*	Yunnan, China	KIZ 049025	KX811931
98	*Xenophrysglandulosa*	Yunnan, China	KIZ 048439	KX811762

Two male advertisement calls were recorded using a SONY ICD-TX50 recorder at an ambient temperature of approximately 21 °C. Acoustic analysis was performed using Raven Pro 1.6 (Cornell Laboratory of Ornithology, Ithaca, NY, USA) following the method outlined by Köhler et al. (2017). The protocol involved calculating audio-spectrograms with a fast-Fourier transform (FFT) of 512 points, 50% overlap, and 172 Hz grid-spacing, using Hanning windows. Refer to Suppl. material 1: table S3 for comparison data on bioacoustics.

## ﻿Results

### ﻿Molecular analyses

**Figure 2.**
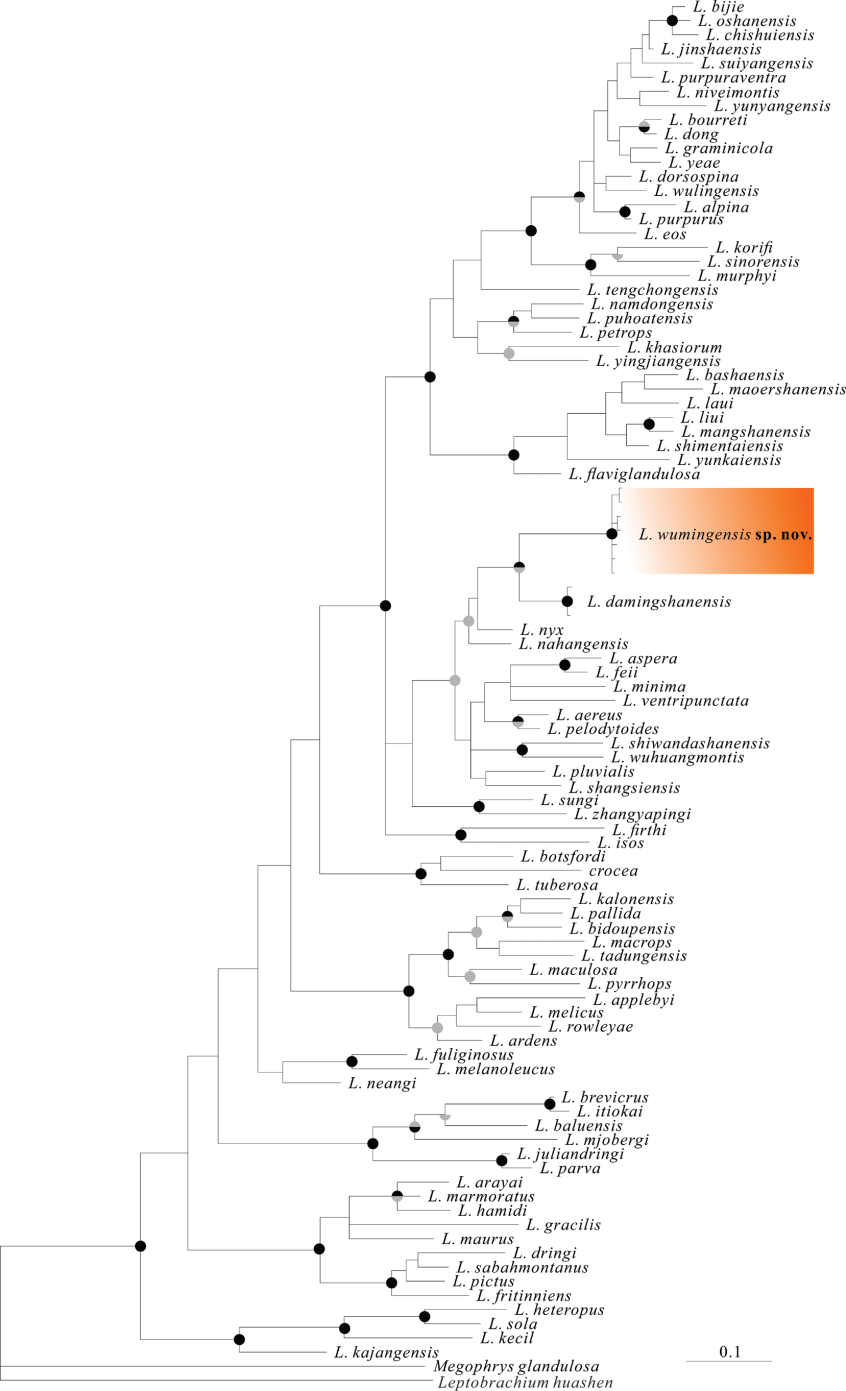
BI trees based on the 16S mtDNA fragments. Node support is indicated on branches as Bayesian posterior probabilities (upper half; >0.95 = grey, 1 = black) and maximum likelihood support (lower half; >70%<90% = grey, >90% = black).

The molecular analyses conducted using BI and ML methods produced similar topologies, as depicted in Fig. 2. However, the phylogenetic relationships of *Leptobrachella* species showed weak internal node support and remained unresolved (Fig. 2). The phylogenetic trees indicated that the DMS specimens are closely related to *L.damingshanensis*, *L.nahangensis* and *L.nyx*. Furthermore, the genetic divergence of the 16S gene fragment between the DMS specimens and all other available homologous sequences of *Leptobrachella* species was found to be greater than 7.1% for the analyzed fragment (Suppl. material 1: table S4).

### ﻿Bioacoustics

**Figure 3.**
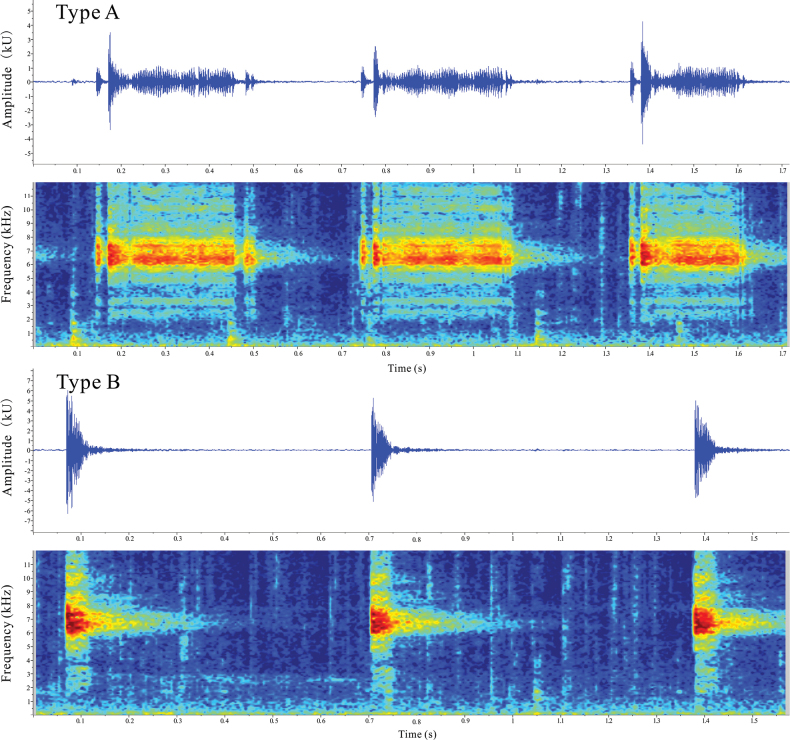
Advertisement calls spectrograms of *L.wumingensis* sp. nov.

The calls of two individuals were recorded in the field (NNU 01058 and NNU 01086). Two types of advertisement calls (Type A and Type B; Fig. 3) were detected. The call durations of Type A ranged from 0.2454 s to 0.4306 s, with an average of 0.3407 ± 0.0423 s, while the call intervals of Type A ranged from 0.1784 s to 0.3756 s, with an average of 0.2364 ± 0.0333 s. For Type B, the call durations ranged from 0.0416 s to 0.0903 s, with an average of 0.0605 ± 0.0094 s, and the call intervals ranged from 0.3700 s to 1.7740 s, with an average of 0.8932 ± 0.3810 s. The dominant frequencies were observed to be between 6.0–7.5 kHz (at 21.0 °C). The DMS specimens exhibited two types of calling models and relatively high dominant frequencies, which distinguishes them from the known advertisement callings in the genus *Leptobrachella* (Suppl. material 1: table S3).

### ﻿Morphological characters

The DMS specimens can be readily differentiated from other species in the same phylogenetic clade. For instance, adult males of *L.damingshanensis* have a significantly larger body size (SVL 33.6–34.4 mm) and possess a pair of distinct tangerine glands on the proximal thigh area, as well as rudimentary webbing and narrow lateral fringes on their toes. In contrast, the DMS specimens have indistinct tangerine glands on the proximal thigh area, and no toe webbing or lateral fringes.

Taken together, molecular data, acoustic analyses and morphological characters support the conclusion that the DMS specimens represent a distinct and previously unrecognized species of *Leptobrachella*, which is described below.

### ﻿Taxonomic account

#### ﻿ Leptobrachella wumingensis sp. nov.

Taxon classificationAnimaliaAnuraMegophryidae

D1E78D64-CBEB-520C-B54F-FA2ED125CE5C

https://zoobank.org/2FC88888-1A4C-4042-93E1-FE7F52A27AF7

Figs 4 , 5

##### Type materials.

***Holotype*.**NNU 01058, adult male, collected at the Damingshan National Nature Reserve, Wuming District, Nanning City, Guangxi, China (23.507°N, 108.395°E; elevation 1214 m), collected by Wei-Cai Chen on 12 April 2023. ***Paratypes*.**NNU 201907009, NNU 01086, two adult males, collected at the same locality as the holotype; NNU 201907009 collected by Gui-Dong Yu on 23 May 2019, NNU 01086 collected by Wei-Cai Chen on 14 April 2023; NNU 00283–6, four adult females collected at the same locality as the holotype on 16 June 2021 by Wei-Cai Chen; NNU 01059–60, two adult females collected at the same locality as the holotype on 12 April 2023 by Wei-Cai Chen.

##### Etymology.

The specific name ‘*wumingensis*’ is derived from the type locality, Wuming District, Nanning City, Guangxi, China. The proposed common name in English is Wuming Leaf Litter Toad, and in Chinese, it is called Wu Ming Zhang Tu Chan (武鸣掌突蟾).

##### Diagnosis.

*Leptobrachellawumingensis* sp. nov. is classified under *Leptobrachella* based on specific morphological features, including its relatively small body size, presence of an inner metacarpal tubercle, macro-glands on the supra-axillary and femoral glands, lack of vomerine teeth, and a whitish vertical bar on the anterior tip of the snout, according to previous studies (Dubois 1983; Lathrop et al. 1998; Delorme et al. 2006; Matsui 2006). *Leptobrachellawumingensis* sp. nov. can be differentiated from other species in its genus by a combination of the following characters: (1) medium size (SVL 26.0–26.7 mm in males, 30.6–34.8 mm in females); (2) absence of toe webbing and lateral fringes; (3) shagreened and granular dorsal surface; (4) pale brown dorsum with darker brown markings; (5) iris bicolored, with the upper half copper and fading to silver in the lower half; (6) presence of small irregular black spots and tangerine tubercles on the flanks; and (7) two types of advertisement callings and high dominant frequencies.

##### Description of holotype.

**Figure 4.**
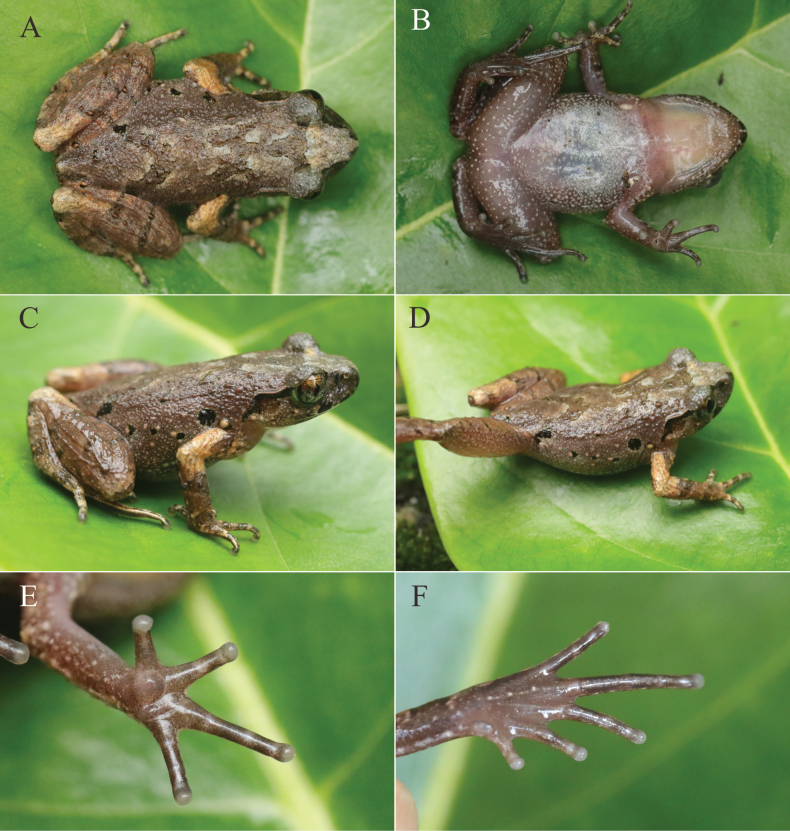
Morphological characters of *L.wumingensis* sp. nov. (NNU 01058) **A** dorsal view **B** ventral view **C, D** lateral view **E** ventral view of hand **F** ventral view of foot.

Head length almost equal to width (HW/HL = 1.02); snout bluntly rounded in profile and dorsal view, projecting slightly over lower jaw; nostril oval-shaped, closer to tip of snout than eye; canthus rostralis distinct; loreal region distinctly sloping, slightly concave; pupil vertical; eye diameter less than snout length (ED/SNT = 0.96); tympanum distinct and rounded, diameter about 49% that of eye; vomerine teeth absent; tongue with a deep notch at posterior tip; supratympanic fold distinctly raised from corner of eye to the posterior of tympanum (Fig. 4A–D).

Tips of fingers slightly swollen; relative finger lengths I < II < IV < III; subarticular tubercles absent; prominent inner palmar tubercle, small outer palmar tubercle; finger webbing and dermal fringes absent; nuptial pad on fingers absent. Tips of toes rounded, slightly swollen; relative toe lengths I < II < V = III < IV; subarticular tubercles absent, replaced by dermal ridges; prominent and elongated inner metatarsal tubercle; outer metatarsal tubercle absent; toe webbing and lateral fringes absent. Tibia 48% of SVL; tibiotarsal articulation reaching middle of eye; heels meeting when hindlimbs flexed at right angles with respect to body (Fig. 4E, F).

Dorsal surface shagreened and granular; upper eyelid with small tubercles; ventral surface without tubercles; flanks with sparse tubercles; pectoral glands elongated, approximately 2.1 mm in diameter; small femoral glands oval, approximately 0.6 mm in diameter, closer to knee than to vent; supra-axillary glands oval, approximately 0.8 mm in diameter; ventrolateral glandular line discrete; ventral surface of thigh with some tubercles (Fig. 4A–D).

##### Color in life.

Dorsum light brown with brown markings, an inverted triangle marking between eyes, an irregular brown marking on scapular region, a brown ‘Λ’ marking on rear of dorsum; tympanum pale brown; supratympanic fold black from the posterior corner of eye to the anterior of supra-axillary glands; two wide black bars on upper lip; five irregular black spots and several small light tangerine tubercles on flanks; transverse brown bars on dorsal surface of hindlimbs; upper arms light orange; belly with tiny creamy white spots; throat creamy white with tiny light brown spots; lower jaw with creamy white tubercles; ventral surfaces of limbs with sparse creamy white tubercles; pectoral and femoral glands creamy white, supra-axillary glands light orange; pupil black; iris bicolored, upper half copper, fading to silver in lower half (Fig. 4A–D).

##### Color in preservative.

The dorsum and limb surfaces are faded to uniform light brown. Irregular black spots on flanks and bars on limbs are darkish brown. The throat, chest, and belly are creamy white, and pectoral, femoral, supra-axillary, and ventrolateral glands are also creamy white.

##### Variation.

**Figure 5.**
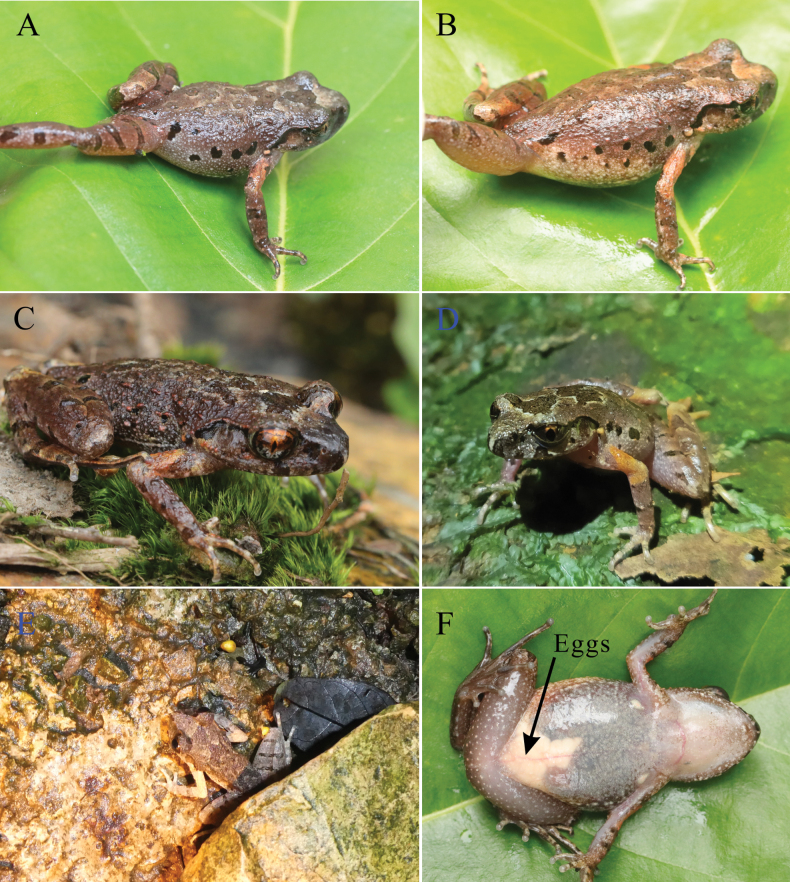
**A** dorsolateral view of NNU 01086 **B** dorsolateral view of NNU 01059 **C** dorsolateral view of NNU 00285 **D**NNU 201907009 in habitat (photo taken *in situ*) **E**NNU 00283 in habitat (photo taken *in situ*) **F** eggs (NNU 01059).

The measurements of the type series are shown in Table 2. The holotype and paratypes exhibit similar color patterns. Females are larger than males in terms of body size. The number of black spots on the flanks varies, ranging from five to eight (Fig. 5A, B). Some individuals have more tubercles on their flanks and their dorsum are rougher compared to the holotype (Fig. 5C).

**Table 2.** Measurements of voucher specimens of *L.wumingensis* sp. nov. (mm). Abbreviations defined in text.

	NNU 01058	NNU 01086	NNU 201907009	NNU 00283	NNU 00284	NNU 00285	NNU 00286	NNU 01059	NNU 01060
Sex	Male	Male	Male	Female	Female	Female	Female	Female	Female
SVL	26.7	26.6	26.0	31.2	31.5	30.6	31.9	34.8	31.6
HL	8.7	8.7	8.9	10.2	10.0	10.2	10.8	10.4	11.2
HW	8.9	9.0	9.3	10.5	10.6	10.8	10.8	11.0	11.2
SNT	3.4	3.8	3.7	4.3	4.2	4.1	4.0	4.5	4.3
ED	3.2	3.6	3.4	4.3	4.8	4.6	4.1	3.7	3.6
IOD	3.0	2.7	2.8	3.1	3.3	3.6	3.3	3.4	2.9
IN	2.7	2.6	2.2	3.4	3.5	3.6	3.4	3.2	3.0
TD	1.6	1.8	1.8	2.4	2.4	1.9	2.1	2.1	2.0
TED	0.9	1.0	0.8	1.3	1.3	1.1	1.4	1.1	0.9
ML	6.2	5.8	6.1	7.3	7.3	7.6	7.4	7.3	7.2
FLL	11.5	11.9	11.4	14.3	14.5	14.4	14.4	13.8	14.4
THL	12.3	11.7	12.9	14.9	16.3	15.4	15.6	14.1	14.0
TIB	12.7	12.8	12.8	14.4	15.9	15.4	15.9	14.9	15.2
PL	11.5	11.4	11.4	13.4	14.4	14.0	14.2	13.6	13.7
FEM	0.6	0.7	0.7	1.5	1.5	1.4	1.2	1.1	1.0

##### Ecology and distribution.

*Leptobrachellawumingensis* sp. nov. inhabits evergreen forests in the DMS at elevations of 1000–1200 m. All specimens were discovered over 10 m away from rocky streams (Fig. 5D, E). The species’ advertisement calls were heard on the rocks in mid-April, and creamy white eggs were found on females’ abdomens in April (Fig. 5F). We dissected four females collected in June and found no eggs in their abdomens. We speculate that the breeding period of the new species is between April and May. Since 2019, we have surveyed over ten rocky streams similar to the type locality but failed to find more sites where the new species occurs. The population of the new species is very rare; we have visited the type locality more than ten times since 2019, but only encountered nine adults. Although both *L.wumingensis* sp. nov. and *L.damingshanensis* occur in the DMS, the two species do not inhabit the same streams, and the closest site where both species are found is 10 km away. Currently, *L.wumingensis* sp. nov. is only known to occur in the DMS (Fig. 1).

##### Comparisons.

To begin with, *L.wumingensis* sp. nov. is distinguished from *Leptobrachella* species found south of the Isthmus of Kra, Malay Peninsula by the presence of supra-axillary and ventrolateral glands (vs absent in the latter). Furthermore, *Leptobrachellawumingensis* sp. nov. and *L.damingshanensis* are sympatric. *Leptobrachellawumingensis* sp. nov. can be differentiated from *L.damingshanensis* by several characters, including smaller body size in males (SVL 26.0–26.7 mm vs 33.6–34.4 mm); indistinct orange glands on the proximal thigh area (vs a pair of distinct tangerine glands on the proximal thigh area); pale brown dorsal skin with brown markings and a shagreened and granular dorsal surface (vs rough dorsal skin with sparse tangerine tubercles and some short longitudinal ridges); the absence of toe webbing and lateral fringes on toes (vs rudimentary toes webbing and narrow lateral fringes on toes); distinct transverse dark brown bars on the dorsal surface of hindlimbs (vs indistinct transverse dark brown bars on the dorsal surface of hindlimbs); and different bioacoustics, including two types of calling models and dominant frequency at 6.0–7.5 kHz (vs a single calling model and dominant frequency at 4.6–5.2 kHz) (Fig. 4, Suppl. material 1: table S3).

According to the phylogenetic analysis, *L.wumingensis* sp. nov., *L.damingshanensis*, *L.nahangensis*, and *L.nyx* constitute a monophyletic group (Fig. 2). In terms of morphology, *L.wumingensis* sp. nov. can be differentiated from *L.nahangensis* by its notably smaller body size in males (SVL 26.0–26.7 mm vs SVL 40.8 mm); lack of toe webbing (vs rudimentary toes webbing); pale brown dorsum with brown markings, a triangle-shaped marking between the eyes, an irregular brown marking on the scapular region, and a brown ‘Λ’ marking on the posterior dorsum (vs dorsum covered with irregular, diffuse dark gray and black spots); and iris that is bicolored, with the upper half copper and the lower half fading to silver (vs uniformly gold iris). Similarly, *L.wumingensis* sp. nov. differs from *L.nyx* in lacking toe webbing (vs rudimentary toes webbing); pale brown dorsum with brown markings, a triangle-shaped marking between eyes, an irregular brown marking on the scapular region, and a brown ‘Λ’ marking on the posterior of dorsum (vs dorsum being greyish brown with dark regularly set rounded spots); and flanks with small, irregular black spots and tangerine tubercles (vs flanks with poorly distinct spots).

In comparison to other recognized *Leptobrachella* species from north of the Isthmus of Kra, *L.wumingensis* sp. nov. is distinguishable from smaller species such as *L.applebyi* (19.6–22.3 mm in males), *L.ardens* (21.3–24.7 mm in males), *L.aspera* (22.4 mm in male), *L.feii* (21.5–22.8 mm in males), *L.korifi* (22.7 mm in female), *L.melica* (19.5–22.7 mm in males), *L.murphyi* (23.2–24.9 mm in males), *L.niveimontis* (22.5–23.6 mm in males) and *L.pluvialis* (21.3–22.3 mm in males) due to its larger size (26.0–26.7 mm in males). Additionally, *L.wumingensis* sp. nov. is distinct from the notably larger *L.sungi* (48.3–52.7 mm in males) and *L.zhangyapingi* (45.8–52.5 mm in males).

*Leptobrachellawumingensis* sp. nov. can be distinguished from *L.aerea*, *L.alpina*, *L.dong*, *L.eos*, *L.firthi*, *L.graminicola*, *L.isos*, *L.khasiorum*, *L.laui*, *L.liui*, *L.murphyi*, *L.purpurus*, *L.shimentaina*, *L.tamdil*, *L.yingjiangensis*, *L.yunkaiensis* and *L.zhangyapingi* by the absence of lateral fringes on its toes (vs. wide lateral fringes); from *L.aspera*, *L.bashaensis*, *L.bidoupensis*, *L.bijie*, *L.botsfordi*, *L.bourreti*, *L.chishuiensis*, *L.damingshanensis*, *L.dorsospina*, *L.feii*, *L.flaviglandulosa*, *L.fuliginosa*, *L.jinshaensis*, *L.jinyunensis*, *L.korifi*, *L.mangshanensis*, *L.maoershanensis*, *L.niveimontis*, *L.pelodytoides*, *L.petrops*, *L.puhoatensis*, *L.purpuraventra*, *L.shangsiensis*, *L.sinorensis*, *L.suiyangensis*, *L.sungi*, *L.tengchongensis*, *L.ventripunctata*, *L.verrucosa*, *L.wuhuangmontis*, *L.wulingensis*, *L.yeae*, and *L.yunyangensis* by the absence of lateral fringes on its toes (vs. narrow lateral fringes). *Leptobrachellawumingensis* sp. nov. is also differentiated from *L.aerea*, *L.botsfordi*, *L.crocea*, *L.graminicola*, *L.eos*, *L.firthi*, *L.isos*, *L.pallida*, *L.petrops*, and *L.tuberosa* by the presence of black spots on its flanks (vs. absent). *Leptobrachellawumingensis* sp. nov. differs from *L.applebyi*, *L.bidoupensis*, *L.kalonensis*, *L.melica*, *L.minima*, *L.nahangensis*, and *L.tadungensis* by the presence of shagreened and granular dorsal surface (vs smooth).

Lastly, *L.wumingensis* sp. nov. differs from other *Leptobrachella* species in terms of acoustic features such as relatively high dominant frequencies and two distinct types of callings (Fig. 3, Suppl. material 1: table S3).

## ﻿Discussion

Based on morphological characters, molecular data and bioacoustics, we have identified the DMS specimens as a new species. The validation of this assignment is supported by significant genetic divergence (>7.1%), high dominant frequencies, complicated calling styles and substantial morphological characters. The DMS reserve is located in the central region of Guangxi, and previous fieldwork by many investigation teams did not discover any *Leptobrachella* species until we reported the first one in 2021 (Chen et al. 2021b). We have conducted annual amphibian surveys in the reserve since 2019 and have found that the population of *L.wumingensis* sp. nov. is rare, with only nine individuals found along with *L.damingshanensis*. Further field surveys are required to determine their distributions for conservation purposes.

According to previous research and our new data (AmphibiaChina 2023; Frost 2023), at least ten *Leptobrachella* species occur in Guangxi, including *L.alpina*, *L.bourreti*, *L.damingshanensis*, *L.liui*, *L.maoershanensis*, *L.shangsiensis*, *L.shiwandashanensis*, *L.ventripunctata*, *L.wuhuangmontis* and *L.wumingensis* sp. nov. Six of these species have been described in the past five years. The weak dispersal abilities and forest-dependent niches of *Leptobrachella* species may contribute to underestimating their diversity and distribution in Guangxi. Thus, more surveys are needed to understand the true species diversity and distribution of *Leptobrachella* in the region.

## Supplementary Material

XML Treatment for Leptobrachella wumingensis

## ﻿Additional information

### Author ORCIDs

Wei-Cai Chen https://orcid.org/0000-0002-2398-4079

Wan-Xiao Peng https://orcid.org/0000-0001-5635-9061

Peng Li https://orcid.org/0000-0001-8311-0544

Gui-Dong Yu https://orcid.org/0009-0008-1928-6594

### Data availability

All of the data that support the findings of this study are available in the main text or Supplementary Information.

## Supplementary materials

Supplementary material 1 Supplementary information https://doi.org/10.3897/zookeys.1178.106038.suppl1 Data type morphological, phylogenetic Explanation note **table S1**: Literature for morphological characters for species of the genus *Leptobrachella*; **table S2**: Comparative material examined; **table S3**: Dominant frequency of advertisement calls of species available in the genus *Leptobrachella*; **table S4**: Uncorrected *p*-distance in *Leptobrachella* species based on 16S mtDNA fragments. https://binary.pensoft.net/file/899702This dataset is made available under the Open Database License (http://opendatacommons.org/licenses/odbl/1.0/). The Open Database License (ODbL) is a license agreement intended to allow users to freely share, modify, and use this Dataset while maintaining this same freedom for others, provided that the original source and author(s) are credited.Wei-Cai Chen, Wan-Xiao Peng, Peng Li, Gui-Dong Yu
